# Diversities in Virulence, Antifungal Activity, Pigmentation and DNA Fingerprint among Strains of *Burkholderia glumae*

**DOI:** 10.1371/journal.pone.0045376

**Published:** 2012-09-18

**Authors:** Hari S. Karki, Bishnu K. Shrestha, Jae Woo Han, Donald E. Groth, Inderjit K. Barphagha, Milton C. Rush, Rebecca A. Melanson, Beom Seok Kim, Jong Hyun Ham

**Affiliations:** 1 Department of Plant Pathology and Crop Physiology, Louisiana State University Agricultural Center, Baton Rouge, Louisiana, United States of America; 2 Rice Research Station, Louisiana State University Agricultural Center, Rayne, Louisiana, United States of America; 3 Division of Biotechnology, College of Life Sciences and Biotechnology, Korea University, Seoul, Republic of Korea; University of the West of England, United Kingdom

## Abstract

*Burkholderia glumae* is the primary causal agent of bacterial panicle blight of rice. In this study, 11 naturally avirulent and nine virulent strains of *B. glumae* native to the southern United States were characterized in terms of virulence in rice and onion, toxofalvin production, antifungal activity, pigmentation and genomic structure. Virulence of *B. glumae* strains on rice panicles was highly correlated to virulence on onion bulb scales, suggesting that onion bulb can be a convenient alternative host system to efficiently determine the virulence of *B. glumae* strains. Production of toxoflavin, the phytotoxin that functions as a major virulence factor, was closely associated with the virulence phenotypes of *B. glumae* strains in rice. Some strains of *B. glumae* showed various levels of antifungal activity against *Rhizoctonia solani*, the causal agent of sheath blight, and pigmentation phenotypes on casamino acid-peptone-glucose (CPG) agar plates regardless of their virulence traits. Purple and yellow-green pigments were partially purified from a pigmenting strain of *B. glumae*, 411gr-6, and the purple pigment fraction showed a strong antifungal activity against *Collectotrichum orbiculare*. Genetic variations were detected among the *B. glumae* strains from DNA fingerprinting analyses by repetitive element sequence-based PCR (rep-PCR) for BOX-A1R-based repetitive extragenic palindromic (BOX) or enterobacterial repetitive intergenic consensus (ERIC) sequences of bacteria; and close genetic relatedness among virulent but pigment-deficient strains were revealed by clustering analyses of DNA fingerprints from BOX-and ERIC-PCR.

## Introduction

*Burkholderia glumae* is a seed-born rice pathogen that causes bacterial panicle blight (BPB), which is an emerging major disease problem in many rice-producing areas around the world, including the southeastern United States and South and Central American countries [1]. In particular, significant yield losses from BPB were experienced in rice-producing areas of the southeastern United States, including Louisiana, Texas and Arkansas in 1996, 1997, 2000, and most recently, in 2010 [2]. *Burkholderia gladioli* also causes BPB but tends to be less virulent and less prevalent than *B. glumae* [3]. Prolonged high night temperatures and frequent rainfalls at the rice heading stage are thought to be important environmental predispositions for outbreaks of this disease [4], [5].

BPB is a problematic disease not only because it causes severe economic damages, but also because there are few effective methods to control this disease. Oxolinic acid used as a seed treatment or foliar application is the only chemical that can control BPB, but it is not commercially available in the United States [2]. Additionally, the occurrence of oxolinic acid-resistant strains of *B. glumae* can limit the use of this chemical [1], [6]. Low nitrogen usage, which may reduce disease severity, has not been successful, and early planting, which may avoid high temperatures during the heading stage, can be useless if high temperatures are reached early in the season [2]. Growing disease resistant varieties would be the best option, but only partially resistant varieties that lack desired commercial characteristics are available [1], [7], [8].

Multiple virulence factors are involved in the bacterial pathogenesis of *B. glumae*. Molecular genetic studies performed by several research groups identified major pathogenic determinants of *B. glumae*, including the phytotoxin, toxoflavin [9], [10], and lipase [11]. Bacterial motility mediated by flagella is also required for the pathogenicity of *B. glumae* [12]. Toxoflavin and lipase production as well as the bacterial motility mediated by flagella are controlled by a quorum-sensing system composed of a LuxI-family acyl-homoserine lactone (AHL) synthase, TofI, and a LuxR-family AHL receptor, TofR [9], [11], [12]. Additional virulence factors known to contribute to the full virulence of *B. glumae* include the PehA and PehB polygalacturonases [13], the KatG catalase [14], and the Hrp type III secretion system (T3SS) [15].

More than 300 *B. glumae* strains were previously isolated from rice plants with symptoms of BPB growing in rice fields in Louisiana and other states in the southeastern United States, including Texas and Arkansas [3]. In this previous study, some of the isolated strains showed asymptomatic or hypovirulent phenotypes on both rice sheaths and rice panicles in greenhouse tests [3]. In this study, 11of the 19 strains that showed asymptomatic phenotypes in preliminary virulence tests were confirmed to be naturally avirulent after a series of rigorous virulence assays in the greenhouse and in the field. These eleven naturally avirulent strains were tested with nine virulent strains of *B. glumae* for various phenotypes including virulence on onion bulb scales, toxoflavin production, pigmentation on CPG agar media, and antifungal activity, as well as for genetic variations revealed by BOX- and ERIC-PCR analyses.

## Materials and Methods

### Bacterial Strains and Culture Conditions

Bacterial strains and plasmids used in this study are listed in Table 1. *B. glumae* and *Escherichia coli* strains were routinely grown and maintained in Luria Bertani (LB) agar or broth medium [16] at 30 to 37°C.

**Table 1:** Bacterial strains used in this study.

Strain name	Description/Origin	Reference
*Burkholderia glumae*		
106sh-5	Avirulent, non-pigmenting/U.S. (Louisiana)	[3]
106sh-9	Avirulent, non-pigmenting/U.S. (Louisiana)	[3]
11sh2-2-a	Virulent, pigmenting/U.S. (Louisiana)	[3]
117g1-7-a	Virulent, non-pigmenting/U.S. (Louisiana)	[3]
189gr-8	Virulent, pigmenting/U.S. (Texas)	[3]
191sh-1	Virulent, pigmenting/U.S. (Texas)	[3]
201sh-1	Virulent, pigmenting/U.S. (Louisiana)	[3]
237gr-5	Avirulent, pigmenting/U.S. (Louisiana)	[3]
257sh-1	Avirulent, pigmenting/U.S. (Louisiana)	[3]
336gr-1	Virulent, non-pigmenting/U.S. (Louisiana)	[3]
366gr-2	Avirulent, non-pigmenting/U.S. (Arkansas)	[3]
261gr-9	Virulent, pigmenting/U.S. (Louisiana)	[3]
961149-4-4	Avirulent, pigmenting/U.S. (Louisiana)	[3]
395-2	Avirulent, non-pigmenting/U.S. (Arkansas)	[3]
379gr-1-b	Avirulent, non-pigmenting/U.S. (Arkansas)	[3]
396gr-2	Avirulent, non-pigmenting/U.S. (Arkansas)	[3]
411gr-6	Virulent, pigmenting/U.S. (Arkansas)	[34]
957856-41-c	Virulent, pigmenting/U.S. (Louisiana)	[3]
98gr-1	Avirulent, non-pigmenting/U.S. (Louisiana)	[3]
99sh-7	Avirulent, non-pigmenting/U.S. (Louisiana)	[3]
ATCC33617	Avirulent, non-pigmenting/Japan (type strain)	[28]
AU6208	Virulent, pigmenting/U.S. (Michigan)	[38]
BGR1	Virulent, non-pigmenting/South Korea	[29]
LSUPB223	A *toxA*::pKNOCK_Gm_ derivative of 336gr-1, Nit^R^, Gm^R^	This study
*Burkholderia gladioli*		
ATCC51989	An ATCC strain of *B. gladioli*	[39]
*Escherichia coli*		
HB101	F^–^ *thi*-1 *hsd20* (r^–^_B_m^–^_B_) *sup E44 recA13 ara-14 leuB6 proA2 lacY1 rpsL*20 (Sm^R^) *xyl*-5 *mtl*-1	[40]
S17-1 λpir	*recA thi pro hsdR* (res– mod+)(RP4::2-Tc::Mu-Km::Tn*7*) λ *pir* phage lysogen	[23]
Plasmids and Mutants		
pKNOCK_Gm_	A suicide vector, R6K *ori*, Gm^R^	[22]
pKNOCK_Gm_::ToxA-int	A clone of *toxA* internal region in pKNOCK_Gm_, Gm^R^	This study
pRK2013::Tn*7*	ColE1 *mob* ^+^ *tra*_RK2_ *Δrep*_RK2_ *repE kan*::Tn*7* (Tp^R^, Sm^R^, Sp^R^ )	[41]
pSC-A-amp/kan	A PCR cloning Vector, Ap^R^, Km^R^	Agilent Technology (Santa Clara, CA, USA)

### Recombinant DNA Techniques

Procedures for routine DNA cloning and amplification were conducted according to Sambrook *et al*. (2001).

### Confirmation of *B. Glumae*

Diagnostic PCR for *B. glumae* was conducted with the species specific primer sets, 5′-ACACGGAACACCTGGGTA-3′ and 5′-TCGCTCTCCCGAAGAGAT-3′ for the 16S-23S rDNA internal transcribed spacer (ITS) region and 5′-GAAGTGTCGCCGATGGAG-3′ and 5′-CCTTCACCGACAGCACGCAT-3′ for *gyrB*, using the previously described reaction conditions for each primer set [3], [17].

### Virulence Assay on Rice Plants

The rice variety Trenasse, which is highly susceptible to BPB, was used for testing the virulence of *B. glumae* strains on rice panicles. Overnight cultures of *B. glumae* strains on LB agar plates were resuspended in sterile tap water at a concentration of ca. 1×10^8^ CFU/ml (OD_600_ = 0.1). Rice plants at the 20 to 30% heading stage were inoculated twice with a two-day interval between sprays (∼ 2 ml/plant). Disease symptoms were evaluated 14 d after the first inoculation. The experiments were conducted in the greenhouse and in the field during the 2009 growing season. For field experiments at the LSU AgCenter Rice Research Station (Crowley, Louisiana, USA), the susceptible variety Trenasse was grown in rows (12 to 15 plants per row) with ca. one-foot intervals between rows. A row of the partially resistant variety Jupiter was grown between every four rows of Trenasse. Virulence was scored using a 0–9 scale in which 0 indicated no symptoms and 9 indicated more than 80% discolored panicles. Overall disease severity of an entire row was scored with four replications for each treatment. For greenhouse experiments, rice plants were grown in plastic pots (15 cm diameter by 20 cm height) containing a soil mixture of clay, Jiffy Mix® (Ferrry-Morse Seed Co, Fulton, KY, USA) and sand in a 3∶1:1 ratio. Rice plants were inoculated with the same method used for field experiments. Greenhouse tests for determining the virulence phenotypes of *B. glumae* strains were repeated three times. In each test, disease severity of each rice plant was scored for each treatment with four replications. Yield reduction caused by *B. glumae* infection was determined with the yield data obtained from field experiments. Rice grains from a single row infested with each strain or mock-inoculated with water were collected, dried to 13% moisture, and weighed. Four replications were implemented in the yield data.

### Onion Assay

Yellow onions used in this study were purchased from a local market. The virulence of each *B. glumae* strain on onion bulb scales was tested following a previously developed method with minor modifications [18]. Briefly, 5 µl of bacterial suspension containing ca. 5×10^5^ CFU in 10 mM MgCl_2_ was applied to a ca. 2 mm-wound on the inner surface of an onion bulb scale made with micropipette tip. Inoculated onion scales were incubated in a wet-chamber at 30°C and virulence was determined by measuring the macerated area on each onion bulb scale after 48 h.

### Determination of Toxoflavin Production and Pigmentation

To determine the production of toxoflavin by each *B. glumae* strain, *B. glumae* cells were streaked on a King’s B (KB) agar plate [16] and incubated for 24 h at 37°C. Toxoflavin production was determined based on the presence of a yellow pigment diffused from the bacterial colonies into the surrounding agar medium. To determine the pigmentation phenotype of each *B. glumae* strain, bacterial cells were streaked on a CPG agar plate [16] and incubated for 48 h at 30°C.

### Measurement of Antifungal Activity Against *Rhizoctonia solani*

Antifungal activities of *B. glumae* strains against *R. solani* were measured following a previously reported method [19] with some modifications. Briefly, one ml of an overnight culture of each *B. glumae* strain was centrifuged, washed twice with fresh LB broth, and resuspended in 100 µl of fresh LB broth. Ten-microliter aliquots of each suspension were pipetted onto three locations around the center of a potato dextrose agar (PDA) plate. Inoculated PDA plates were incubated overnight (∼ 16 h) at 37°C. Mycelial plugs 5 mm in diameter were cut from *R. solani* cultures grown on PDA at 30°C and placed in the center of each PDA plate containing three spots of *B. glumae*. The length of the inhibition zone between *B. glumae* and *R. solani* was measured for each bacterial spot 48 h after incubation at 25°C. Nine replications were performed for each strain of *B. glumae*.

### BOX- and ERIC-PCR and Cluster Analyses of the DNA Fingerprints

BOX- and ERIC-PCR were conducted following a previously established method [20]. PCR products were separated on a 1.8% agarose gel run at 60 V for 18 h and were visualized with a Kodak Gel Logic 1500 imaging system (Rochester, NY, USA). DNA fingerprints of individual strains generated from BOX- and ERIC-PCR were converted into a binary matrix by scoring amenable DNA bands as present or absent. Cluster analyses of the DNA fingerprints were performed with unweighted pair-group method of averages (UPGMA) using MEGA5 [21].

### Generation of the *toxA* Toxoflavin Deficient Mutant, LSUPB223

An internal region of *toxA* was amplified using the primers, 5′-TTTCGGGCGTGAAATCTATC-3′ and 5′-AGCGGTAGAAGCTGAACTGG-3′. The amplified PCR product was cloned into the PCR cloning vector, pSC-A-amp/kan, using a StrataClone PCR Cloning Kit (Agilent Technologies, Santa Clara, CA, USA) following the manufacturer’s instructions. The insert of the resultant PCR clone, pSC::ToxA-int, was digested with the *Kpn*I and *Sac*II restriction enzymes and then ligated to the *Kpn*I/*Sac*II-cut pKNOCK_Gm_ suicide vector [22], to generate pKNOCK_Gm_::ToxA-int (Table 1). *Escherichia coli* S17-1 λpir [23] was used to maintain the pKNOCK vectors and pKNOCK_Gm_::ToxA-int. *E. coli* HB101, which carries the helper plasmid, pRK2013 [24], was used in triparental mating to introduce pKNOCK_Gm_::ToxA-int into a virulent strain of *B. glumae*, 336gr-1. *toxA* mutants resulting from homologous recombination with the vector containing the internal fragment of *toxA* were selected on LB agar containing nitrofurantoin and gentamycin. The toxoflavin producing phenotypes of these mutants were verified on KB agar plates.

## Results

### Determination of Naturally Avirulent Strains of *B. glumae* Isolated from Rice Fields in the Southeastern United States

**Figure 1:**
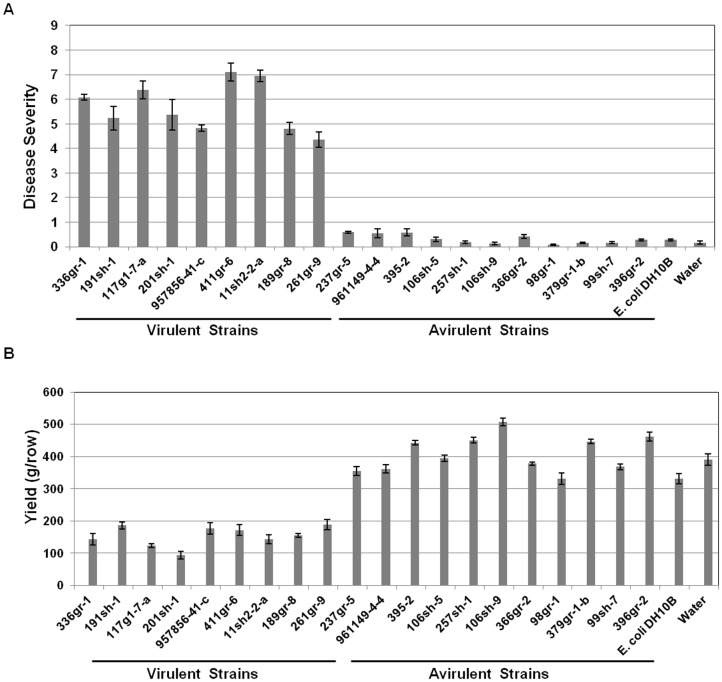
Virulence effects of *Burkholderia glumae* strains on rice panicles. A) Disease severities of rice panicles inoculated with virulent and avirulent strains of *B. glumae*. Disease scales: no symptom, 0; 1–10% symptomatic area, 1; 11–20% symptomatic area, 2; 21–30% symptomatic area, 3; 31–40% symptomatic area, 4; 41–50% symptomatic area, 5; 51–60% symptomatic area, 6; 61–70% symptomatic area, 7; 71–80% symptomatic area, 8; more than 80% symptomatic area, 9. B) Rice yields (g/16-foot row) of plants inoculated with virulent and avirulent strains of *B. glumae*. These data were obtained from the susceptible rice variety, Trenasse, and error bars indicate the standard deviation from four replications.

In a previous study, some strains of *B. glumae* isolated from rice fields in Louisiana and other southeastern states failed to produce symptoms on both rice sheaths and panicles [3]. In this study, 19 out of 24 strains that showed asymptomatic phenotypes in the previous tests were confirmed by diagnostic PCR with species specific primers that anneal to the ITS region or *gyrB* of *B. glumae* (data not shown) to be *B. glumae.* These 19 strains were re-examined for their virulence in rice along with a highly virulent strain of *B. glumae*, 336gr-1, and *Escherichia coli* DH10B as a positive and negative control, respectively. From repeated tests in the greenhouse (data not shown) and in the field, 11 of the 19 strains of *B. glumae* were confirmed to be avirulent, whereas the remaining eight strains were determined to be virulent (Figure 1A). In addition, the 11 avirulent strains did not cause significant yield reductions when compared to the negative control, while virulent strains including the virulent reference strain, 336gr-1, caused 50 to 75% yield reductions (Figure 1B).

### Test of Onion Bulb Scales as an Alternative for Efficient and High-throughput Virulence Assays for Strains of *B. glumae*

**Figure 2:**
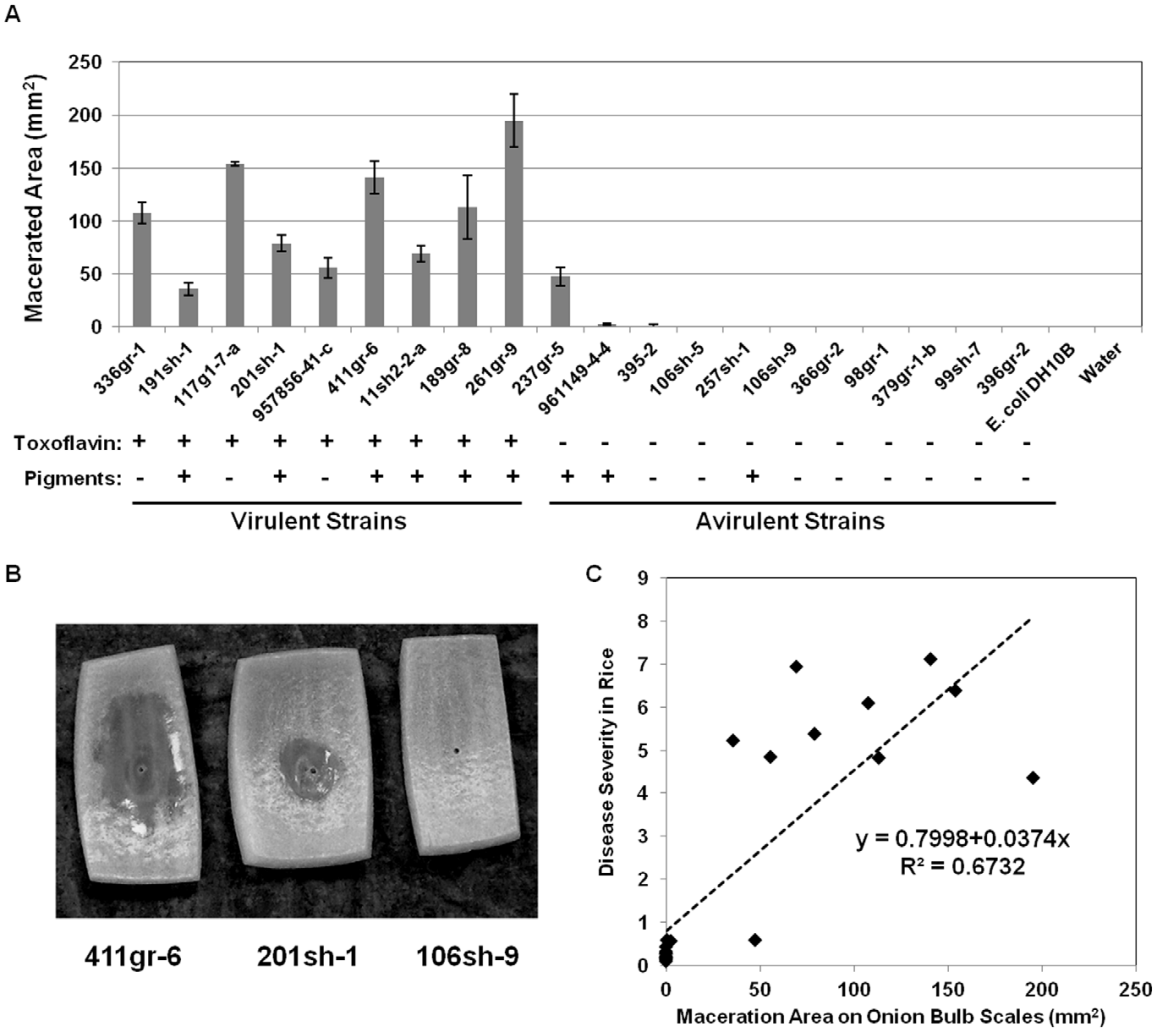
Virulence of *Burkholderia glumae* strains on onion bulb scales. A) Area of macerated tissue of onion bulb scales inoculated with virulent and avirulent strains of *B. glumae*. Error bars indicate the standard deviation from three replicates. Phenotypes in toxoflavin production and pigmentation are indicated under the name of each strain. B) Onion bulb scales showing various levels of maceration after 48 h incubation at 30°C after inoculation with a highly virulent, a moderately virulent, and an avirulent strain of *B. glumae*, 411gr-6, 201sh-1, and 106sh-9, respectively. C) Correlation between the abilities of *B. glumae* strains to cause maceration on onion bulb scales and to produce symptoms on rice panicles: The dotted line represents a linear regression line. All the parameter estimates were statistically significant at α <0.001 indicating strong relationship between the disease severity in rice panicles and the macerated area on onion scales. Statistical analysis was performed by using SAS 9.3 version.

Remarkably, all of the virulent strains of *B. glumae* that were tested caused maceration of the onion bulb scale tissue, but all of the avirulent strains of *B. glumae* except 237gr-5 did not cause maceration of the onion bulb scale tissue (Figures 2A and 2B). Moreover, the virulence of *B. glumae* on onion bulb scales was highly correlated with that on rice panicles (R^2^ = 0.6732) (Figure 2C).

### Production of Toxoflavin

**Figure 3:**
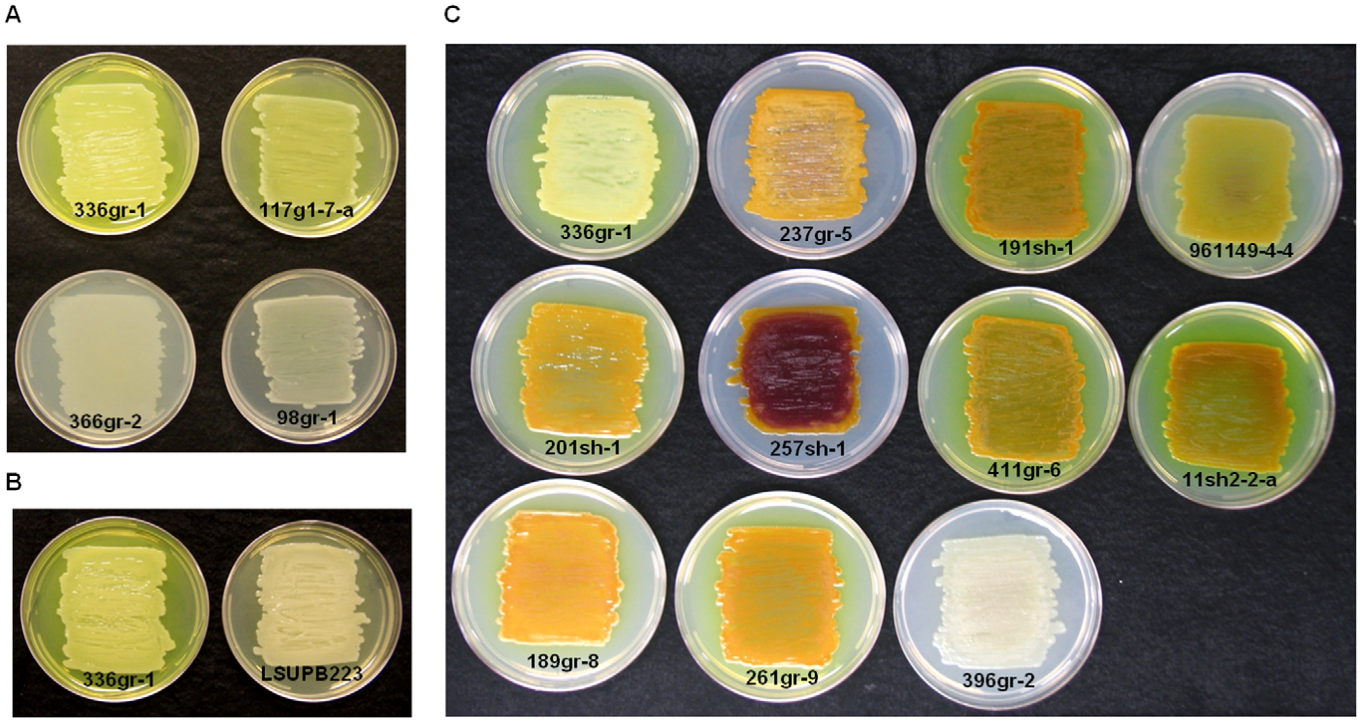
Toxoflavin and pigmentation phenotypes of strains of *B. glumae*. A) Toxoflavin production on KB agar plates by virulent strains (336gr-1 and 117g1-7-1) and avirulent strains (366gr-2 and 98gr-1) of *B. glumae*. B) Toxoflavin production by a virulent strain of *B. glumae*, 336gr-1, and its *toxA* mutant derivative, LSUPB223. C) Pigmentation of strains of *B. glumae* on CPG agar plates in comparison with a non-pigmenting virulent strain of *B. glumae*, 336gr-1, and a non-pigmenting avirulent strain of *B. glumae*, 396gr-2. The bright yellow pigment of strain 336gr-1 is toxoflavin. Photos were taken after 24 h incubation at 37°C for A) and B) and after 48 h incubation at 30°C for C).

Toxoflavin is a bright yellow pigment that has antibiotic and phytotoxic activity and that is produced by and functions as a major virulence factor of *B. glumae* [9], [10], [25]. A virulent strain of *B. glumae*, 336gr-1, produced a yellow pigment in culture media, including LB and KB. In solid media, this yellow pigment is diffused from bacterial colonies into the surrounding agar medium (Figure 3A). Mutation of *toxA*, a gene required for toxoflavin biosynthesis [9], [10], abolished the production of this yellow pigment (Figure 3B). In addition, the yellow pigment extracted from the culture media with chloroform showed absorbance maxima at 258 and 393 nm (data no shown) like a recent study on toxoflavin [26]. These results together indicate that the yellow pigment is toxoflavin and that the production of toxoflavin can be determined by the presence of the yellow pigment in media with cultures of *B. glumae*. As shown in Figure 2A, all of the *B. glumae* strains that were virulent to rice produced toxoflavin, whereas all of the strains that were avirulent to rice did not, indicating that toxoflavin production is closely associated with the virulence of *B. glumae* to rice. However, one toxoflavin-deficient strain, 237gr-5, produced maceration symptoms on onion bulb scales, indicating that toxoflavin is dispensable for the maceration activity of *B. glumae* in onion.

### Production of Yellow-green and Purple Pigments in CPG Agar Medium

Nine of the 20 strains tested produced unknown pigments when grown on CPG agar plates (Figures 2A and 3C). These pigments were easily distinguished from toxoflavin, which is bright yellow and diffused into the agar medium from bacterial colonies. Six of the nine virulent strains and three of the 11 avirulent strains produced pigments (Figures 2A and 3C). Partial purification of the pigments produced by the pigmenting strain, 411gr-6, yielded three fractions with different colors; yellow-green, purple, and brown (Figure S1A). The brown fraction (Figure S1A) is likely a mixture of multiple unknown substances, not a single material (data not shown). Thus, the observed pigmentation may be due to at least two different pigments, which were yellow-green and purple (Figures 3C and S1A). The purple pigment was restricted to the bacterial colonies, whereas the yellow-green pigment was diffused into the agar medium (Figure 3C). The yellow-green pigment purified from agar extract exhibited strong green fluorescence when illuminated with UV light with UV absorption maxima at 202, 238, 301, 448 and 464 nm. The purple pigment exhibited a strong antifungal activity (Figure S1B). In terms of color and antifungal activity, the purple pigment resembles the virulence factor of the opportunistic human pathogen *Pseudomonas aeruginosa*, pyocyanin, which is also known as “blue phenazine” [27]. However UV absorbance spectra and spectroscopic data including ^1^H-NMR indicated that the purple pigment is different from pyocyanin (data not shown). It is noteworthy that a diverse range of phenotypic variation in pigmentation was observed among the pigment-producing strains (Figure 3C). Especially, 257sh-1 produced excessive amounts of the purple pigment compared to the other pigment-producing strains (Figure 3C). In addition, three virulent strains, 191sh-1, 411gr-6 and 11sh2-2-a, produced larger amounts of the diffusible yellow-green pigment compared to other *B. glumae* strains (Figure 3C). Unlike toxoflavin, the pigmentation did not appear to be related to bacterial pathogenesis in onion (Figure 2A).

### Antifungal Activities of Naturally Avirulent Strains of *B. glumae* Against *Rhizoctonia solani*

**Figure 4:**
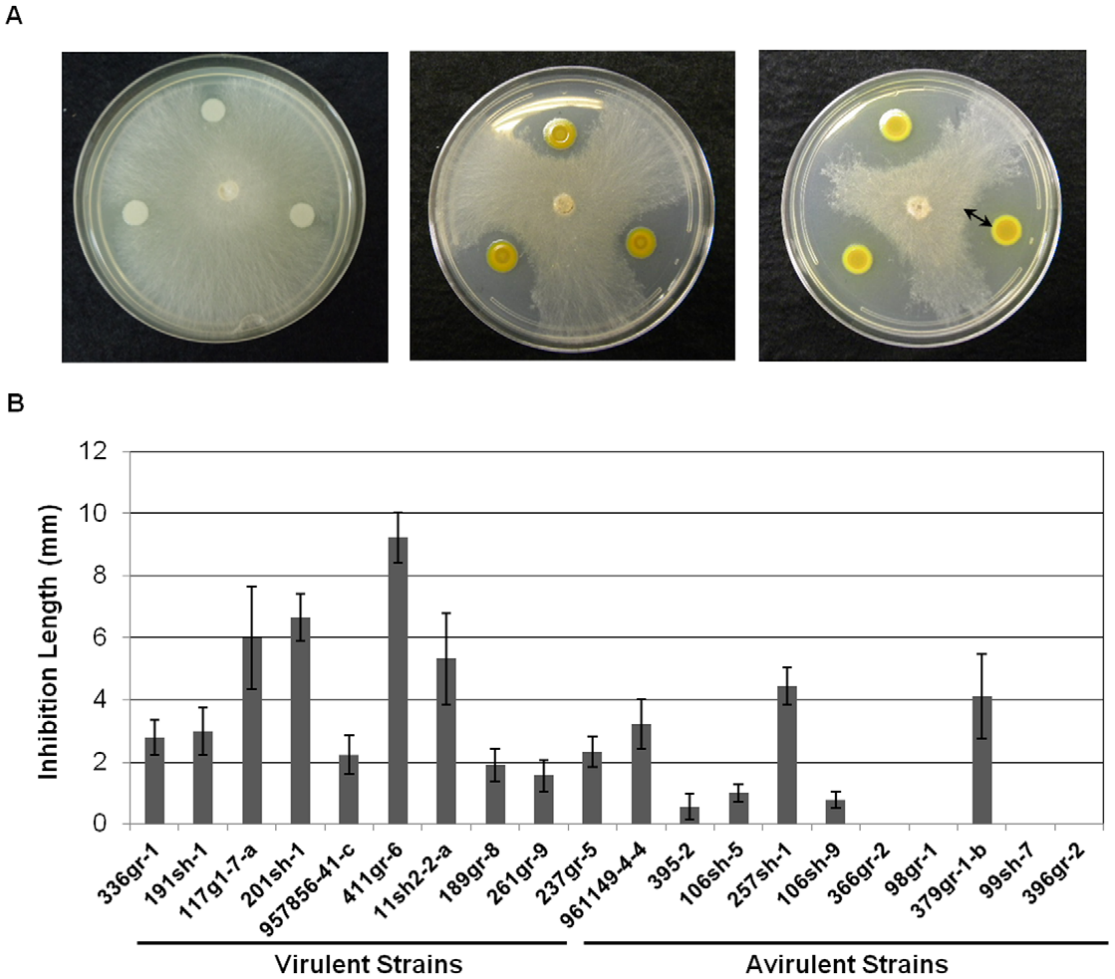
Antifungal activities of *Burkholderia glumae* strains against *Rhizoctonia solani*. A) Various levels of antifungal activities against *R. solani* after 48 h incubation at 25°C. *B. glumae* in the center of each plate was incubated overnight (∼ 16 h) prior to the inoculation of *R. solani*. The arrow indicates the measurement used to quantify the antifungal activity. B) Antifungal activities of virulent and avirulent strains of *B. glumae* determined by the length of the inhibition zone between the edge of the colony of *B. glumae* and the edge of the mycelial growth of *R. solani*. Error bars indicate the standard deviation from nine replications.

All of the virulent strains of *B. glumae* showed observable antifungal activities, whereas only seven of the 11 avirulent strains showed observable antifungal activities (Figures 4A and 4B). The highly virulent and toxoflavin- and pigment-producing strain, 411gr-6, showed the highest antifungal activity among all of the strains tested (Figure 4B). An avirulent strain, 379gr-1-b, showed antifungal activity despite its toxofalvin- and pigment-deficient phenotype, indicating that this strain possesses at least one antifungal system that is independent of toxoflavin and pigment production in CPG agar medium (Figure 4B).

### Comparison of Genomic Structures between Virulent and Avirulent Strains of *B. glumae* by BOX- and ERIC-PCR

**Figure 5:**
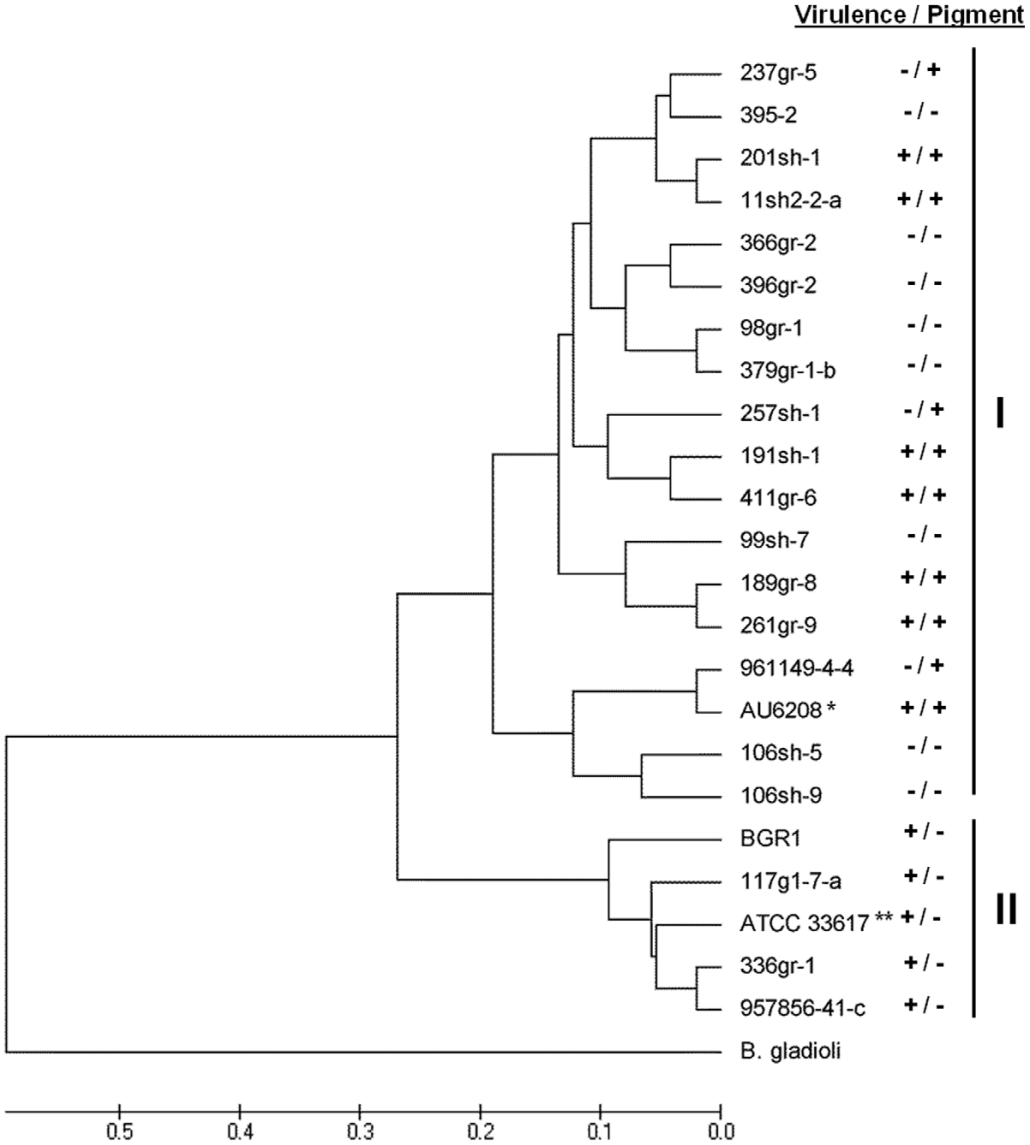
Phylogenetic tree generated from UPGMA analysis of the BOX-PCR fingerprints of *Burkholderia glumae* strains. *: AU6208 was reported to be virulent to rice in a previous study [11] and tested for the pigmentation phenotype by another research group (J. J. LiPuma, *personal communication*). **: The phenotypes of BGR1 were indicated based on the previous studies reported by Kim *et al.* (2004) and Jeong *et al.* (2003). ***: ATCC33617 is the type strain originally isolated as the causal agent of bacterial panicle blight and its lost pathogenicity by a spontaneous mutation of *tofR* could be restored by the addition of a functional copy of *tofR* [11].

**Figure 6:**
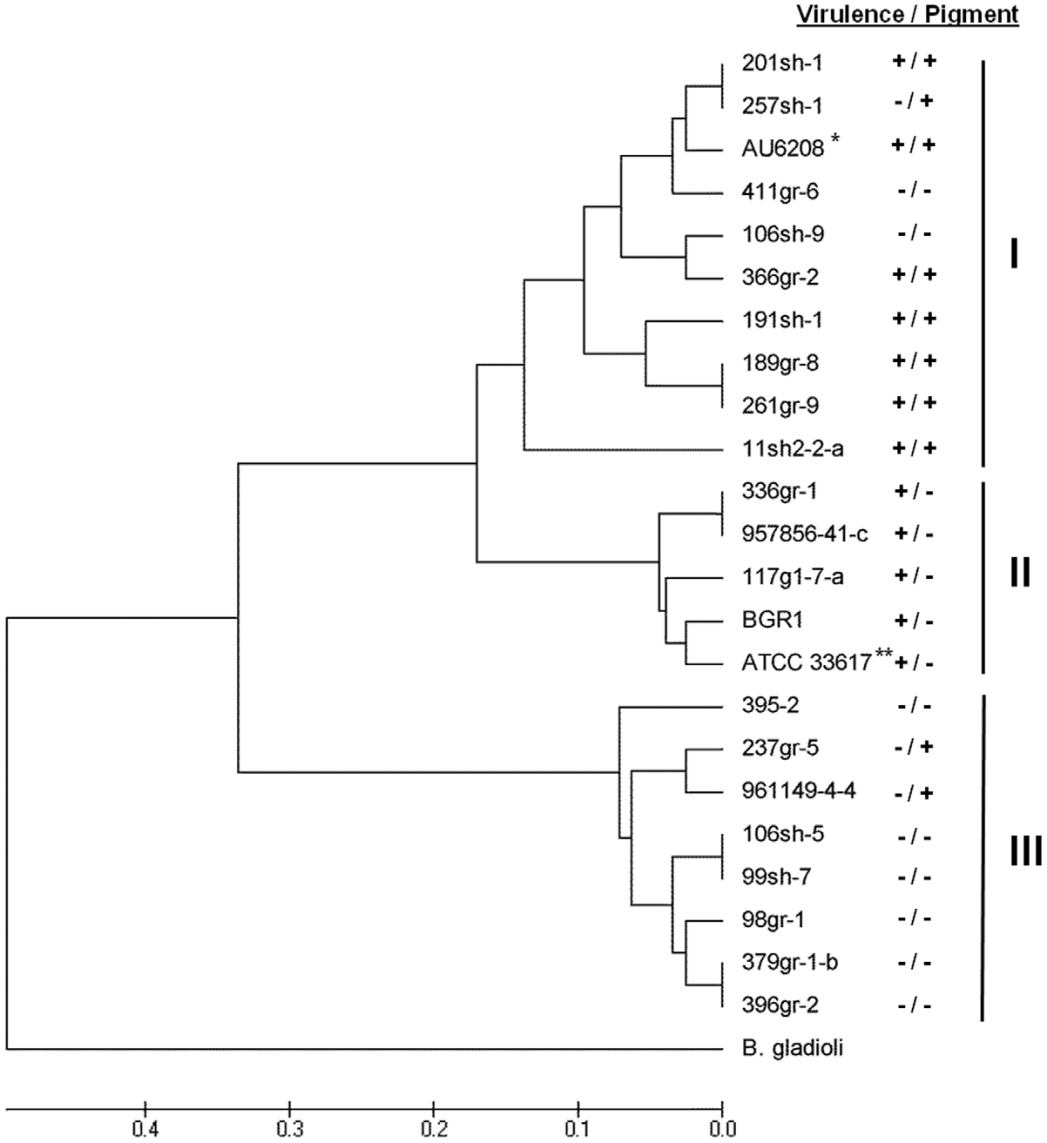
Phylogenetic tree generated from UPGMA analysis of the ERIC-PCR fingerprints of *Burkholderia glumae* strains. *: AU6208 was reported to be virulent to rice in a previous study [11] and tested for the pigmentation phenotype by another research group (J. J. LiPuma, *personal communication*). **: The phenotypes of BGR1 were indicated based on the previous studies reported by Kim *et al.* (2004) and Jeong *et al.* (2003). ***: ATCC33617 is the type strain originally isolated as the causal agent of bacterial panicle blight and its lost pathogenicity by a spontaneous mutation of *tofR* could be restored by the addition of a functional copy of *tofR* [11].

The observed phenotypic variations in virulence-related traits (virulence on rice and onion and toxoflavin production), antifungal activity and pigment production among the strains of *B. glumae* prompted us to investigate the genetic relatedness among strains. BOX- and ERIC-PCR revealed genomic variation among the 23 tested strains of *B. glumae*, which included the 20 U.S. strains as well as the type strain ATCC33617^T^ [28], a Korean strain, BGR1 [29], and a clinical strain, AU6208 [11](Figures 5 and 6). Because only DNA samples were available for BGR1 and AU6208, phenotypes of these strains could not be determined in our laboratory. However, the virulent Korean strain, BGR1, did not produce the dark pigments on CPG agar medium [29], whereas AU6208 was shown to be virulent to rice [11] and to produce pigments on CPG medium (J. LiPuma, personal communication). The genomic DNA of the *B. gladioli* strain ATCC51989 was included as a control and used as an outgroup in phylogenetic analyses.

Various BOX-PCR fingerprints were observed among strains of *B. glumae* (data not shown). Twenty-six band classes ranging from 330 bp to 3,500 bp that were reproducible in two independent reactions were scored as previously described and used to generate a dendogram (Figure 5). The dendogram revealed two major phyletic groups. The dendogram indicated that major phyletic groups were not separated on the basis of virulence or pigmentation (Figure 5). Group I was a polyphyletic group that contained the majority of the *B. glumae* strains, including all the naturally avirulent strains and all the pigment-producing strains. Group II was a polyphyletic group that contained virulent, pigment-deficient strains and the type strain ATCC33617^T^ (Figure 5).

Genetic differences were also observed in ERIC-PCR fingerprints (data not shown). Twenty-one band classes between 130 bp and 3500 bp that were amenable and reproducible in two independent reactions were scored as previously described and used to create a dendogram (Figure 6). This dendogram revealed three major phyletic groups. Group I contained all of the virulent, pigment-producing strains; Group II contained all of the virulent, non-pigmenting strains; and Group III contained only avirulent strains (Figure 6).

Although differences were observed between the BOX and ERIC dendograms, all of the strains that grouped together in Group II in the BOX analysis also grouped together in the ERIC analysis (Figures 5 and 6).

## Discussion

In this study, 11 strains of *B. glumae* indigenous to the southeastern U.S. were confirmed to be naturally avirulent through a series of virulence tests conducted in the field and greenhouse. These naturally avirulent strains did not produce any obvious symptoms of BPB or cause any significant yield reduction compared to the nine virulent strains tested (Figures 1A and B). At least 5% of the *B. glumae* strains isolated from rice fields in the southeastern U.S. showed avirulent phenotypes (data not shown). Because the avirulent strains studied were isolated from rice plants showing BPB symptoms, they are presumably derivatives of a virulent strain that lost their pathogenicityvia spontaneous mutation after host infection. It might also be possible that populations of *B. glumae* present in natural conditions typically contain avirulent cells.

Although it is unknown how avirulent strains of *B. glumae* are generated in nature, this phenomenon may be explained by several possible mechanisms. First, major deletion(s) of genomic DNA could cause a loss of multiple virulence genes. It was recently reported that the cluster of *hrp/hrc* genes encoding the Hrp T3SS is absent in the genome of a naturally avirulent strain of *Pseudomonas syringae*, 508, probably due to a deletion event [30]. Second, a point mutation could occur in a core regulatory gene that globally controls multiple virulence factors or in a gene that encodes an important virulence factor. Indeed, the ATCC strain of *B. glumae* tested in this study, ATCC33617^T^, was reported to be avirulent because of a frameshift mutation in *tofR*, which encodes the TofR AHL receptor that functions as a central regulatory element for the production of major virulence factors of *B. glumae*, including toxoflavin and lipase [11]. In addition, four different natural mutant alleles of *avrBs2*, the avirulence gene corresponding to *Bs2* of pepper in gene-for-gene resistance, were observed in natural strains of *Xanthomonas campestris* that showed attenuated virulence phenotypes in pepper plants with a *bs2/bs2* genetic background [31]. Finally, multiple mutations in a genome could also be responsible for the loss of pathogenicity in nature. To identify the cause(s) of the avirulent phenotypes of *B. glumae* strains from infected rice plants, the expression of known and potential virulence genes and the presence of the functional quorum-sensing system required for the pathogenicity of *B. glumae* in the 11 avirulent strains are currently being studied in our laboratory. Whole genome sequencing and comparison with virulent strains would also help to elucidate the genetic basis of naturally-occurring, non-pathogenic strains of *B. glumae*.

An alternate assay system using onion bulb scales for determining the virulence of *B. glumae* was also developed in this study. This assay system was previously used by Jacobs *et al*. (2008) to test strains of *B. cepacia* and *B. cenocepacia* pathogenic to onion. In our study, virulent strains of *B. glumae* were able to cause symptoms on onion bulb scales and virulence on onion bulb scales was highly correlated with virulence on rice panicles (Figure 2C). Strain 237gr-5 was the only strain of the 20 strains tested, that showed different virulence/avirulence features between two different host systems (avirulent to rice but virulent to onion)(Figures 1 and 2A). Virulence tests on rice are time-consuming, labor-intensive, and require a lot of space. The high correlation observed between the virulence of *B. glumae* in rice and that in onion strongly suggests that onion bulb scales can be used as an excellent alternative assay for convenient and high-throughput virulence tests of *B. glumae* strains and would serve as a powerful tool for large-scale functional genomic studies of this bacterium. Nevertheless, caution should be made when onion bulb scales are used as a surrogate system for determining the virulence of *B. glumae* in rice because virulence factors responsible for maceration of onion tissue may be different from those responsible for panicle blighting of rice.

Production of toxoflavin, was closely related to the bacterial virulence phenotypes in rice. All nine virulent strains produced toxoflavin, whereas all 11 avirulent strains did not produce toxoflavin (Figures 1 and 2A). The ability of each bacterial strain to produce toxoflavin was determined by observing the production of the bright yellow pigment released into the culture medium (Figure 3A). Directional mutation of *toxA* via homologous recombination, which resulted in the loss of ability to produce toxoflavin [10], caused the loss of yellow pigment production (Figure 3B), indicating that the yellow pigment is toxoflavin. These facts strongly suggest that naturally avirulent strains of *B. glumae* can be readily determined by their disability to produce toxoflavin in the culture media. Even though the pathogenicity of the *B. glumae* strains in rice was tightly linked to their ability to produce toxoflavin in this study, toxoflavin may not fully account for the pathogenesis of *B. glumae* because mutation of a toxoflavin synthesis gene could not completely abolish bacterial virulence in previous studies [10](Shrestha and Ham, unpublished). Since all of the avirulent strains produced very weak observable symptoms (Figure 1A), it is likely that these strains are defective in the production of multiple virulence factors that are collectively required for full virulence. In fact, it has been shown that *B. glumae* produces additional virulence factors, including lipase [11], the Hrp T3SS [15] and polygalacturonases [13], which contribute to the virulence of this pathogen to rice. Production of known and potential virulence factors of *B. glumae* by individual avirulent strains is currently being analyzed (Karki and Ham, unpublished).

It is intriguing that some strains of *B. glumae* produce unique pigments in CPG medium (Figure 3C). Diverse variations in the pigmentation phenotype were observed within the pigment-producing strains of *B. glumae* (Figure 3C). Two different pigments (yellow-green and purple) were partially purified from a pigmenting strain, 411gr-6 (Figure S1), and their chemical and physical properties were also partially determined (data not shown). They were easily distinguished from the bright yellow toxoflavin. To the best of our knowledge, this pigmentation phenomenon of *B. glumae* has not been reported elsewhere. It was reported that some strains of *B. cenocepacia* produced melanin-like pigments in tyrosine-enriched media and that those pigments may act as scavengers of reactive oxygen species generated from oxidative burse responses of host cells [32]. Later, it was found that the melanin-like pigment produced by a clinical strain of *B. cenocepacia* was likely to be a pyomelanin synthesized from a homogentisate (HGA) through the action of 4-hydroxyphenylpyruvic acid dioxygenase (HppD), and that pigment production was abolished by disruption of *hppD* [33]. However, mutation of *hppD* in 411gr-6, a pigment-producing strain of *B. glumae*, did not change the pigmentation phenotype of this strain, suggesting that the pigments produced by *B. glumae* on CPG agar medium are different from the melanin-like pigments produced by *B. cenocepacia* (data not shown). We recently found that a two-component regulatory system, composed of the PidS sensor histidine kinase and the PidR response regulator, is essential for the production of all three pigments in *B. glumae* [34]. In addition, all of the pigment-deficient mutants screened from random Tn*5*-mutangenesis of the pigment-producing strain 411gr-6 were deficient in the production of all three pigments, implying that they may be synthesized via a common regulatory and biosynthetic pathway [34].

Antifungal activities were detected from most of the strains tested in this study (Figure 4B). In particular, all of the virulent strains showed various antifungal activities (Figure 4B). It is probable that the observed antifungal activities of the virulent strains may be conferred in part, if not fully, by toxoflavin, which is known to have a broad toxic effect on prokaryotes and eukaryotes [35]. However, some avirulent strains that do not produce toxoflavin also showed antifungal activities, indicating that these avirulent strains produce additional antifungal compounds. Interestingly, the purple pigment from 411gr-6 showed a strong antifungal activity against *C. orbiculare* (Figure S1B) and all the pigmenting avirulent strains also showed high levels of antifungal activities against *R. solani* (Figure 4B), suggesting that the production of this pigment may contribute to the antifungal activities of the pigmenting *B. glumae* strains. The chemical structures of the yellow-green and purple pigments are currently being characterized. Meanwhile, the antifungal activities shown by non-pigmenting avirulent strains including 379-gr-1-b (Figure 4B) indicate the presence of additional antifungal compound(s) produced by some *B. glumae* strains. The avirulent strains with antifungal activities against *R. solani* may be useful tools for biological control of sheath blight and possibly other fungal rice diseases, including blast caused by *Magnaporthe grisea*. We are currently testing the antifungal activities of these strains against other fungal pathogens. Antifungal activities of *Burkholderia* spp. other than *B. glumae* and their application as biological control agents have previously been reported [36], [37]. Nevertheless, naturally avirulent *B. glumae* strains could be a more useful tool for the biological control of phylloplane diseases of rice, including sheath blight and blast, because they could inhibit pathogens in the rice phyllosphere better than other *Burkholderia* spp. that typically persist in the soil and plant rhizophere.

BOX- and ERIC-PCR analyses revealed variations in genome structure among strains of *B. glumae* showing various phenotypic traits and originating from diverse geographic locations (Figures 5 and 6). All of the virulent strains showing pigment-deficient phenotypes were grouped in a single major polyphyletic group in both BOX-and ERIC-PCR phylograms (Group II in both BOX- and ERIC-PCR phylograms) regardless of geographic origin (Figures 5 and 6). Even though the strain ATCC33617^T^, previously known to be an avirulent strain, was grouped together with virulent strains in Group II in both rep-PCR analyses, it would not be unacceptable to consider this strain as a virulent one since it was originally isolated as the causal agent of the rice disease [28] and later lost its ability to perceive the quorum-sensing signal due to a spontaneous point mutation in *tofR* encoding the cognate receptor for the quorum-sensing signal of *B. glumae* and since the production of virulence factors was restored by the introduction of a functional *tofR* clone [11]. In our independent study, all of the avirulent U.S. strains with the exception of 237gr-5 that was virulent to onion (Figure 2A), also showed deficiency in quorum-sensing (Karki and Ham, unpublished). However, unlike ATCC33617^T^, pathogenicity could not be restored by the introduction of a DNA clone carrying both *tofI* and *tofR* genes in these strains, suggesting that mutation on the *tofI/tofR* locus is not the only cause of the avirulent phenotype of the naturally avirulent strains (Karki and Ham, unpublished). It is also noteworthy that all of the virulent pigment-producing strains belonged to Group I in both BOX-PCR and ERIC-PCR analyses, and that Group III from the ERIC-PCR analysis only contained avirulent strains (Figure 6). The clinical strain AU6208, which was previously reported to be virulent to rice [11], was grouped together with the virulent strains producing pigments (Figures 5 and 6). According to the tests by Dr. LiPuma’s research group in University of Michigan, AU6208 also produces pigments on CPG agar plates (J. J. LiPuma, *personal communication*). These cluster analyses from BOX- and ERIC-PCR data and the phenotypic characteristics associated with major polyphyletic groups suggest that multiple lineages of *B. glumae* may exist.

Conclusively, significant phenotypic variations were observed among strains of *B. glumae*, including variation in virulence, pigmentation and antifungal activities. In addition, some phyletic groups based on BOX-and ERIC-PCR fingerprints were associated with virulence and pigmentation phenotypes. Nevertheless, genetic backgrounds of the observed phenotypes, including natural avirulence and pigmentation are still unknown. Comparative genomics approaches with whole genome sequence information, which can now be readily obtained by high-throughput sequencing, would provide important clues to identify the causes of these phenotypic traits. Additional comprehensive population genetics studies should also be conducted to elucidate the genetic lineages of this pathogenic bacterium.

## Supporting Information

Figure S1 **Partially purified pigments of** ***Burkholderia glumae*** **411gr-6 (A) and antifungal activity of the partially purified purple pigment (B).** Peak 4 from high pressure liquid chromatography showed purple color and an antifungal activity against *Collectotrichum orbiculare*. The photo of the antifungal activity was taken 48 h after incubation at 28°C.
(TIF)
