# Supplementary figures and images for: Diversities in Virulence, Antifungal Activity, Pigmentation and DNA Fingerprint among Strains of Burkholderia glumae
Source: PLoS One. 2012 Sep 18;7(9):e45376. doi: 10.1371/journal.pone.0045376 (PMC3445519; doi:10.1371/journal.pone.0045376)

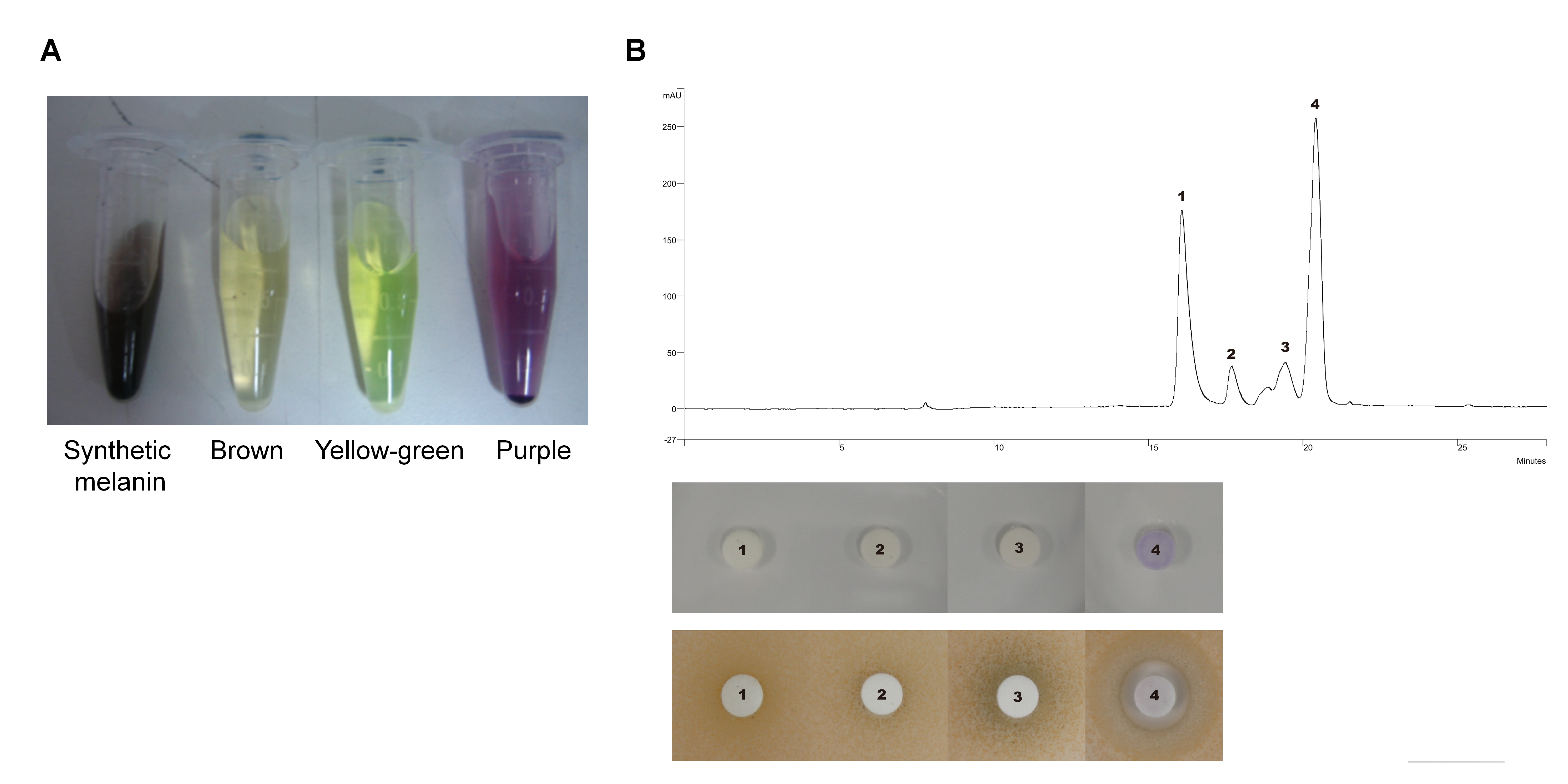

Supplement: Figure S1 — Partially purified pigments of Burkholderia glumae 411gr-6 (A) and antifungal activity of the partially purified purple pigment (B). Peak 4 from high pressure liquid chromatography showed purple color and an antifungal activity against Collectotrichum orbiculare. The photo of the antifungal activity was taken 48 h after incubation at 28°C. (TIF) [file pone.0045376.s001.tif]
